# Pilot study: is there an influence of lower limb positioning during magnetic resonance imaging on muscle cross section shape assessment in the thigh?

**DOI:** 10.1186/s13104-023-06573-y

**Published:** 2023-11-08

**Authors:** Katja Oberhofer, Matthias Blum, Basil Achermann, Silvio R. Lorenzetti

**Affiliations:** 1 Swiss Federal Institute of Sport Magglingen, Performance Sports, Magglingen, Switzerland; 2 Institute for Biomechanics, ETH Zurich, Zurich, Switzerland

**Keywords:** Mri, Musculoskeletal Imaging, Anatomically-based modelling, Muscle shape, Segmentation, J-Index

## Abstract

Positioning in an MRI can influence quantitative measures of the muscle. The goal of this pilot study was to assess the influence of different levels of knee elevation during MRI on the predicted cross-sectional muscle shape in the thigh. Data were acquired in three healthy male participants (age: 29.3 ± 5.1y, height: 181.3 ± 6.4cm, weight: 85.1 ± 3.7kg). For each participant, three MRI scans were taken by a trained radiographer with low, moderate and high knee elevation. The shape of the anatomical cross-sectional areas of the hamstrings and quadriceps in three leg positionings were compared by fitting ellipsoidal functions to the segmented MRI data and calculating the so-called J index for every image slice using the Python scripting language. Different levels of knee elevation resulted in apparent changes in J index for all muscles except vastus medialis. Thereby, the changes were overall more pronounced in the hamstrings compared to the quadriceps. Particularly, by elevating the knee from 8 to 15 degree, the percentage changes in J index were between 7.2 and 13.6% for the hamstrings and between 0.5 and 3.3% for the quadriceps, respectively. For assessing the musculoskeletal properties by means of MRI, a standardized positioning of the leg is required and the knee joint angle should be controlled.

## Introduction

Magnetic resonance imaging (MRI) is the gold standard technique for the assessment of individual musculoskeletal anatomy in medical health and sports science, including muscle hypertrophy and atrophy [1]. Thereby, MRI scans are commonly performed with the subject lying in supine position with straight legs. This position is unspecific to upright activities and could potentially lead to unwanted deformation of the musculature that may bias the accuracy of results. A sub optimal positioning can also influence the assessments; therefore it is important that patients have a suitable positioning in the MRI.

Indeed, previous research showed that cross-sectional area (CSA) measurements of the vastus lateralis by means of ultrasonography significantly changed from lying to standing position; and that moment arms of muscles acting at the hip and the knee joint were significantly different between neutral lying and neutral standing position, thus affecting biomechanical modelling results [2].

The reliable assessment of subject-specific anatomy is further essential for biomechanical analysis of musculoskeletal function and sports performance [3–5] but also for denervation processes. Particularly, subject-specific muscle CSA is correlated to its force production capabilities [6], while three dimensional (3D) muscle shape is known to affect muscle line-of-action, lever arms, joint torque, and thus, the biomechanics of the multi-body musculoskeletal system [2, 5]. Furthermore, muscle volume has been shown to be a major determinant of joint torque [7]; thus, further highlighting the need for accurate subject-specific assessment of musculoskeletal anatomy for biomechanical analysis in sports science.

On the other hand, it was recently found that MRI-based assessment of spinal muscle volume was robust in terms of subject positioning [8]. Yet, spinal muscles are small in size. It is likely that these findings are not applicable to larger and more bulky muscle groups, for example in the thigh, which are affected by larger soft-tissue deformation in lying versus upright standing position.

Building on this research gap, the goal of this pilot study was to assess the influence of different levels of knee elevation during MRI scanning on muscle shape prediction in the thigh. In particular, it was hypothesized that changes in lower extremity positioning during MRI scanning significantly affect the predicted hamstrings and quadriceps cross-sectional shape due to changes in soft-tissue distribution with respect to the imaging plane. The outcome of this pilot study was expected to help inform MRI imaging protocols for subject-specific, anatomically-based modelling towards more accurate analysis of individual physical performance.

## Methods

### Ethical approval

for this pilot study was given by the regional ethics committee. Data were acquired in three healthy male participants (age: 29.3 ± 5.1y, height: 181.3 ± 6.4cm, weight: 85.1 ± 3.7kg). Written consent was obtained from all participants prior to data acquisition. Four stacks of transverse dual-echo 3D gradient images were obtained with a 3 Tesla Siemens Prisma scanner (96 slices each, voxel size = 0.65 × 0.65 × 3.0mm^3^, TR/TE1/TE2 = 3.9/1.23/2.46ms, FOV = 445 × 418mm^2^, fat- and water-separating reconstruction) covering the legs from the upper pelvic crest to the feet.

**Fig. 1:**
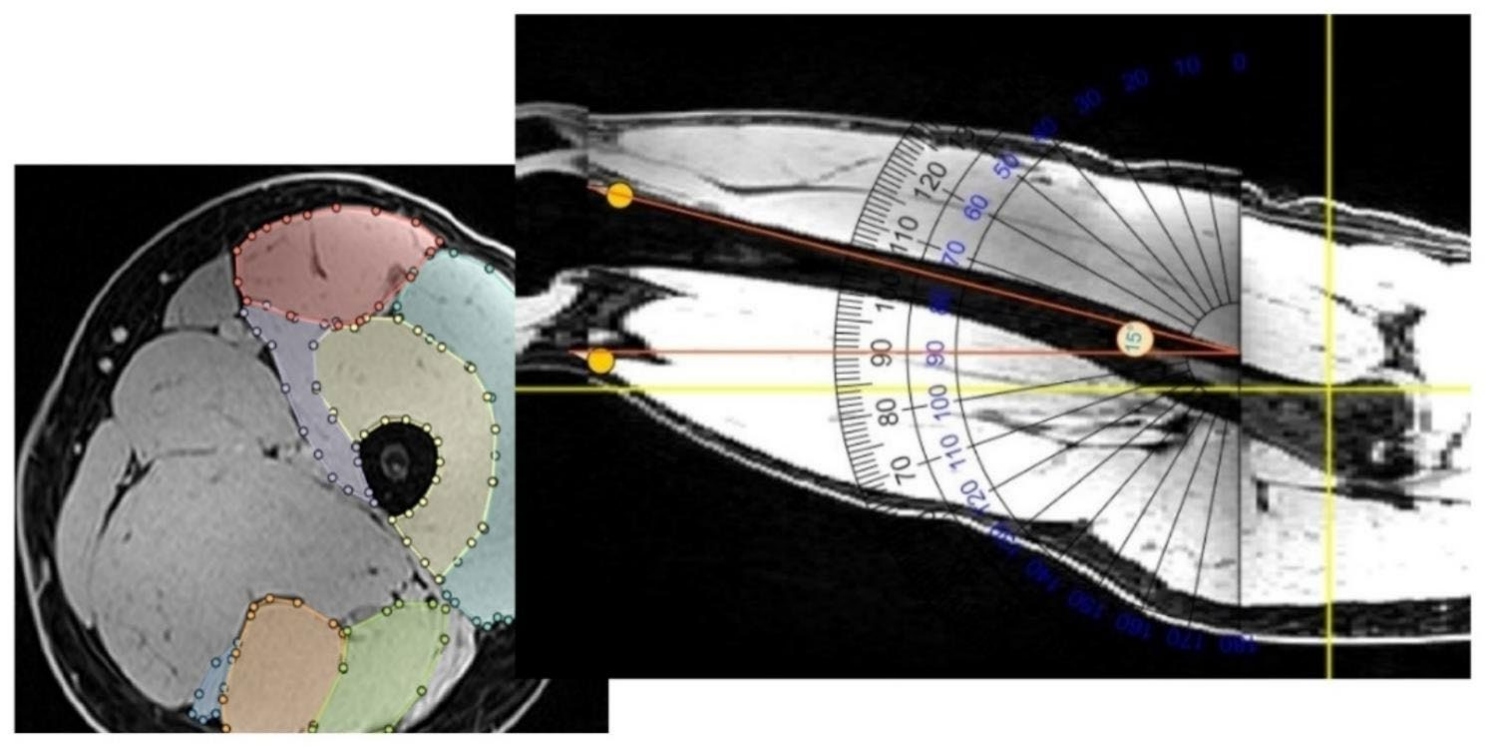
MRI-based segmentation of the cross-sectional shape of the quadriceps and the hamstring muscles in each image slice (left). Digital goniometer to assess the angle between the anatomical axis of femur and the transversal imaging plane (right)

Each participant was scanned three times with different leg positionings. Specifically the knee has been elevation with pillows by a trained radiographer. All the participants were scanned with the same amount of supporting pillows (high, modest, no elevation) and the scans were performed in the same order from high to no elevation. Based on the image data, the anatomical CSA of the following thigh muscles were segmented: vastus lateralis, vastus intermedius, vastus medialis, rectus femoris, semimembranosus, semi-tendinosus and biceps femoris (Fig. 1). For image segmentation, the open-source software SASHIMI (https://github.com/bartbols/SASHIMI), was used. Additionally, the angles between the anatomical axis of femur and the normal vector of the transversal imaging plane were derived using a digital goniometer to quantify knee elevation for the different lower limb positions (Fig. 1).

The cross-sectional muscle shape in every slice was quantified by fitting ellipsoidal functions to the segmented MRI data and calculating the so-called J index using the Python scripting language. The ellipsoidal fitting function is based on calculating the covariance matrix of the point cloud that represents the segmented muscle CSA from MRI. Using the eigenvalues and eigenvectors of the covariance matrix, the ratio between the length and the width of the best fitting ellipse can be calculated and the orientation of the principal axes can be derived. The J index was adapted from a previous study that analyzed and categorized seed shapes in a similar manner [9]. Thereby, the common region that is shared by the fitted ellipsoidal shapes and the segmented MRI data is divided by the region that is not shared between the two. Given the same ellipsoidal reference shape, changes in the average J indexes for different subject positioning allowed to determine whether the cross-sectional muscle shape was affected by different levels of knee elevation, i.e. different soft-tissue distribution with respect to the imaging plane.

## Results

**Fig. 2:**
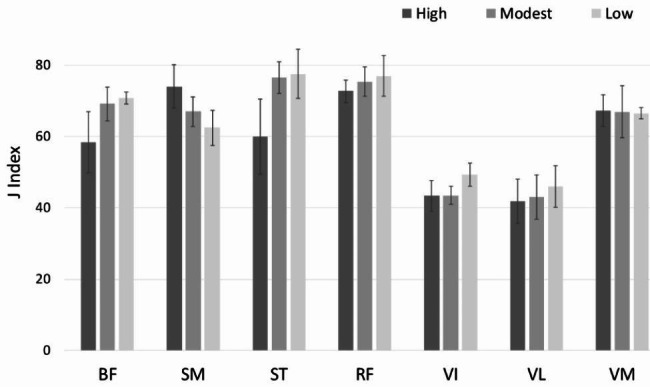
Mean J Index and standard deviation for Biceps Femoris (BF), Semimembranosus (SM), Semitendinosus (ST), Rectus Femoris (RF), Vastus Intermedius (VI), Vastus Lateralis (VL) and Vastus Medialis (VM) during MRI scanning in Position 1 (high knee elevation), Position 2 (moderate knee elevation) and Position 3 (no knee elevation)

The average angles between the normal vector of the transversal imaging plane and the anatomical axis of femur were 15 degree (high), 12 degree (modest) and 8 degree (no elevation). The cross-sectional shape of all muscles except vastus medialis was affected by changes in knee elevation level (Fig. 2). Thereby, the hamstring muscles yielded more obvious changes in J index compared to the quadriceps, which means larger changes in muscle cross-sectional shape for the different levels of knee elevation compared to the ellipsoidal reference shape. By changing knee elevation from 8 to 15 degree, the percentage changes in J index for the hamstrings were 13.6% for semitendinosus, 8.8% for biceps femoris and 7.2% for semimembranosus; while in contrast, the percentage changes for the quadriceps were 3.3% for rectus femoris, 3.0% for vastus intermedius, 1.9% for vastus lateralis and 0.5% for vastus medialis, respectively. Interestingly, the cross-sectional shape of biceps femoris and semitendinosus displayed increasing deviation from the ellipsoidal reference shape with increasing knee elevation, while the opposite was observed for semimembranosus. Vastus medialis was the only muscle for which no apparent change in cross-sectional shape for the different knee elevation levels was observed (Fig. 2).

## Discussion

The results of this pilot study demonstrate that different knee elevation levels (high, modest, no elevation) during MRI scanning has an influence on the assessment of muscle CSA in the thigh. The finding that the hamstrings were more strongly affected by the change in knee positioning than the quadriceps is not surprising, given that they are substantially deformed due to surface contact in supine lying position without knee elevation.

The present study focused on changes in the predicted cross-sectional shape of thigh musculature that are associated with changes in soft tissue distribution with respect to the medical imaging plane. Here, the results confirm and extend previous findings that showed a significant difference in vastus lateralis CSA assessment between lying and standing position [6]. Thereby, Wagle et al. [6] demonstrated that standing measures of muscle shape and size were more strongly correlated to isometric and dynamic force production and may be more accurately assessed using ultrasonography instead of MRI.

In addition to ultrasonography, it has been recently shown that depth camera 3D-imaging systems may be reliable tools for measuring gross thigh volume and shape in standing position [10]. While these advancements in 3D imaging are promising, the derivation of detailed cross-sectional shape of individual muscles from external gross surface measurements remains limited and likely requiring the development of large population-based statistical models for data fitting.

Biomechanical analysis of movement function requires musculoskeletal models that accurately represent subject-specific muscle line-of-action and lever arms to induce joint motion. Here, it is well known that the muscle lines-of-action are dicated by the surrounding soft tissue, which is in turn influenced by gravitational forces and changes in segmental position [2, 5]. Further research is strongly recommended to compare biomechanical analysis results using subject-specific musculoskeletal models based on lying versus standing anatomical measures. In the future, detailed three dimensional imaging can help to overcome some of the current issues.

The weakness of the present pilot study is the low number of participants and limited focus on changes in cross-sectional muscle shape. Further research should be directed to analyse the impact of subject positioning on muscle volume and mass prediction, as well as muscle line-of-action and lever arms, in more subjects and different population groups. Here, preliminary research suggests that muscle volume predictions may also be impacted by changes in subject positioning [6], and that different population groups (e.g. healthy versus pathological muscles) may yield different results [1]. Addtionally, emerging methods to assess subject-specific musculoskeletal anatomy in standing position require further validation, especially when aimed at predicting internal muscle and joint dynamics during functional movement. A further study with more participants would allow to provide a correction score based on the knee flexion angle. A second limitation of this study is, that the person who did the segmentation was not blinded with respect to the knee elevation.

## Conclusion

The reliable assessment of subject-specific musculoskeletal anatomy is essential for biomechanical analysis of movement function in medical health and sports science. By elevating the knee from 8 to 15 degree in the present work, the resulting percentage changes in J index were found to be between 7.2 and 13.6% for the hamstrings and between 0.5 and 3.3% for the quadriceps, respectively. Further in-depth studies of muscle shape changes and associated changes in key biomechanical parameters in more subjects and different lower limb positioning are needed to draw statistically valid conclusions. Yet, it is advisable to carefully consider subject positioning when assessing muscle anatomy by means of MRI, in particular when aiming to derive muscle CSA, line-of-action and lever arms for biomechanical analysis, and further research in this direction is recommended.

## Data Availability

The dataset generated during the current study is available in the figshare repository, doi 10.6084/m9.figshare.23560761.
